# Clinical Associate students’ perception of the educational environment at the University of the Witwatersrand, Johannesburg

**DOI:** 10.4102/phcfm.v7i1.778

**Published:** 2015-04-13

**Authors:** Abigail Dreyer, Audrey Gibbs, Scott Smalley, Motlatso Mlambo, Himani Pandya

**Affiliations:** 1 Centre for Rural Health, Department of Family Medicine, Faculty of Health Sciences, University of the Witwatersrand, South Africa; 2 Faculty of Health Sciences, Department of Paediatrics and Child Health, Division of Community Paediatrics, University of the Witwatersrand, South Africa

## Abstract

**Background:**

An important determinant of a student's behaviour and performance is the school's teaching and learning environment. Evaluation of such an environment can explore methods to improve educational curricula and academic atmosphere.

**Aim:**

To evaluate the educational environment of the Bachelor of Clinical Medicine Practice programme as perceived by students at the University of the Witwatersrand, South Africa.

**Setting:**

This cross-sectional study was conducted with all final-year students (*n* = 25) enrolled in 2011, with a response rate of 88% (*n* = 22). Students were in two groups based in the Gauteng and North-West provinces.

**Methods:**

Data were collected using the Dundee Ready Educational Environmental Measure questionnaire, which was administered to all students. Total and mean scores for all questions were calculated for both groups.

**Results:**

The learning environment was given an average score of 130/196 by the students. Individual subscales show that ‘Academic self-perception’ was rated the highest (25/32), whilst ‘Social self-perception’ had the lowest score (13/24). Positive aspects of the academic climate included: student competence and confidence development; student participation in class; constructive criticism provided; empathy in medical profession; and friendships created. Areas for improvement included: feedback provision to students; course time-tables; ensure non-stressful course; provision of good support systems for students; and social life improvement.

**Conclusion:**

Students’ perceptions of their learning environment were ‘more positive’ than negative. Results from this study will be used to draw lessons for improving the curriculum and learning environment, improve administrative processes and develop student support mechanisms in order to improve their academic experience.

## Abstrait

**Perception des étudiants assistants de clinique de l'environnement éducatif à l'Université du Witwatersrand.**

**Contexte:**

Un déterminant important du comportement et de la performance d’un étudiant est l'environnement scolaire d’enseignement et d’apprentissage. L’évaluation de cet environnement peut permettre de développer des moyens pour améliorer les programmes d’enseignement et l'atmosphère académique.

**Objectif:**

Evaluer l'environnement éducatif du programme de Licence en Pratique de Médecine clinique, tel que le perçoivent les étudiants de l'Université du Witwatersrand, en Afrique du Sud.

**Cadre:**

Cette étude transversale a été faite avec tous les étudiants de dernière année (*n* = 25) inscrits en 2011 avec un taux de réponse de 88% (*n* = 22). Les étudiants étaient dans deux groupes dans les provinces du Gauteng et du Nord Ouest, respectivement.

**Méthodes:**

Les données ont été collectées au moyen du questionnaire de Mesure environnementale éducative de Dundee Ready qui a été distribué à tous les étudiants. On a calculé les notes totales et moyennes de toutes les questions des deux groupes.

**Résultats:**

Les étudiants ont donné une note moyenne de 130/196 à l'environnement d’apprentissage. Les sous-domaines individuels montrent que ‘la perception académique de soi-même’ était la plus élevée (25/32) alors que ‘la perception sociale de soi-même’ avait la note la plus basse (13/24). Les aspects positifs du climat académique comprenaient: le développement de l'assurance et des compétences des étudiants; la participation des étudiants en classe; les critiques constructives formulées; la compassion dans la profession médicale; les amitiés créées. Les domaines à améliorer étaient notamment: les feed-back aux étudiants; l'horaire des cours; assurer des cours non-stressants; donner de bons systèmes de soutien aux étudiants; l'amélioration de la vie sociale.

**Conclusion:**

La perception des étudiants de leur environnement d’apprentissage était ‘plus positive’. Les résultats de cette étude seront utilisés pour tirer des leçons afin d’améliorer le programme scolaire et l'environnement d’apprentissage, de parfaire les processus administratifs et de développer les mécanismes de soutien aux étudiants pour améliorer leur expérience universitaire.

## Introduction

An important determinant of a student's behaviour and performance is the school's teaching and learning environment.^1^ Evidence suggests that a positive learning environment as perceived by students impacts their academic performance and can lead to increased success in both the academic and professional domains.^2^ Innovations in medical curricula (which include a blend of classroom, workplace, clinical and community-based learning) and increasing diversity of the student population in medical courses have led to increased recognition of a need to evaluate the educational environment of medical schools. Evaluation helps to assess if these curricula are beneficial to students and adding to their skills as compared to the traditional counterparts, and to draw lessons for continuous improvement.^3^

The combination of workforce shortages, increasing burden of chronic disease, more treatable conditions, advances in medical technology and an ageing population have led to increasing demands on the healthcare system.^4^ The inequitable distribution of healthcare workers with shortages in rural communities presents a major area of concern for addressing the global burden of diseases and quality healthcare delivery with universal coverage.^5^ Mostly, urban areas show a heavy concentration of healthcare workers whilst the population in rural areas experiences a greater burden of diseases.

As evident from the literature, there have been recommendations in the past that new models of healthcare delivery should be examined to address these issues.^4^ Staffing health facilities with sufficient numbers of appropriately trained health professionals is a major challenge and a prerequisite to implement the National Health Insurance successfully in South Africa.^6^ To fulfil the National Department of Health's vision of ‘Health care for all' it is necessary to develop and employ new health professional cadres to meet the health needs of the population, ensure retention and improve workforce productivity.^7^

Whilst mid-level health workers have been successfully addressing medical workforce shortage in high-income countries such as the United States of America, Europe and Australia,^4,8,9^ they hold great potential for addressing human resources shortages in low- and middle-income countries, as suggested by evidence from mid-level practitioner employment in countries like Uganda, Tanzania, Kenya, Malawi and Mozambique.^10^

With this in mind, South Africa has developed a new cadre of mid-level health professionals called Clinical Associates, with the aim of improving quality of health care at hospitals, revitalising primary health care at district level and universalising health coverage in the country.^10^ Formation of this cadre in South Africa led to development of a 3-year Bachelor of Clinical Medicine Practice (BCMP) degree programme resulting in qualification as a Clinical Associate. The BCMP was started by three universities in South Africa, with the University of the Witwatersrand (Wits) launching its programme in 2009.^11^

The BCMP course structure is based on the principle of developing a sound knowledge of medical and clinical sciences to enable students to understand medical conditions of patients and their management strategies with a patient-centred approach. The curriculum follows an integrated approach with a combination of teaching modalities delivered in the classroom, skills laboratory and district hospitals led by family medicine practitioners and clinical associate tutors.

During this evaluation the third-year BCMP students were placed at various district and provincial hospitals in Gauteng and North-West provinces for clinical rotations. In 2011 the hospitals used in Gauteng included the Kopanong and South Rand District Hospitals and Natalspruit Hospital, whilst those in North-West included Taung District Hospital and Rustenburg and Mafikeng Provincial Hospitals. As per the curriculum, students needed to complete 5-week clinical rotations in the following departments during their third year in 2011: (1) Surgery, (2) Emergency Medicine, (3) Paediatrics, (4) In-patient Medicine, (5) Out-patient Medicine and HIV, and (6) Elective (can choose any department from the above five again or a different department). Students are allowed to remain in one hospital for more than one rotation, generally spending 5–15 weeks at one hospital.

As evident from the literature, many evaluations (qualitative and quantitative) have been conducted globally to measure the academic environment of health sciences programmes (undergraduate and postgraduate) by utilising different methodologies. These evaluations include assessment of medicine, nursing, physiotherapy, dental science, chiropractic and other related programmes.^12^ In order to gather information on whether the programme was meeting the expectations of students in terms of a better learning environment, and if its design was student-centred, an initial evaluation was required to guide course organisers for better development of the programme. It was therefore decided to evaluate the educational environment of the BCMP in 2011 with the rationale of quality improvement, to incorporate students’ feedback in course development and provide a better academic experience for future Clinical Associate students.

This study is a preliminary evaluation which was conducted with the first cohort of third-year students (placed in Gauteng and North-West provinces) who graduated in 2011 as Clinical Associates. Our objectives were firstly to understand and evaluate students’ (Clinical Associates) perceptions and/or experiences of the educational environment in the BCMP programme by using the Dundee Ready Education Environment Measure (DREEM); secondly, to generate a profile of students’ perceptions in terms of the strengths and weaknesses of the educational environment by exploring individual item scores; and lastly, to utilise results to draw lessons for improving the curriculum and learning environment and develop student support mechanisms in order to improve the academic experience.

## Research methods and design

### Study design and setting

This cross-sectional quantitative survey was conducted in 2011 amongst third-year (final-year) Wits BCMP students (based in North-West and Gauteng provinces) who graduated in 2011 as Clinical Associates.

Whilst the overall response rate was 88% (*n* = 22/25), there was an item-specific response rate where items 1, 13, 16 and 28 were responded to by 10 out of 11 students from Gauteng. Although the demographic characteristics of respondents (such as gender and age) were not captured through the questionnaire, we assumed that any differences in these did not have a significant influence on their views about the learning environment of the BCMP. In addition, we assumed that due to small sample size the exclusion of respondents’ demographic information would ensure that their identity was concealed and that no information would be easily identifiable to us.^13^

### Data collection

Data were collected using the self-administered DREEM questionnaire consisting of 50 items to be answered on a 5-point Likert scale ranging from ‘strongly agree’ to ‘strongly disagree’. The questionnaire was administered within a classroom setting after the lecture. Before conducting the study and selecting the instrument we assumed that the learning environment as perceived by students was not restricted to the third year (final-year) of their clinical rotations when they answered the questions, but pertained to the overall environment throughout the three years of the course. We assumed that whilst answering the questions students perceived the terms in the questionnaire as follows:

- ‘Teaching’ – overall teaching including classroom and hospital based;
- ‘Teachers’ – all staff members involved with BCMP including course organisers, lecturers, tutors, clinical supervisors, hospital-based doctors and nurse mentors;
- ‘Atmosphere’ – both classrooms based at Wits as well as clinical based at hospitals in Gauteng and North-West; and
- ‘School'/‘teaching sessions’/‘tutorials’/‘classes’ – pertains to both Wits University and hospitals outside Wits (for clinical rotations).

The universal DREEM tool consists of 50 items with a global score of 200.^14^ Our modified DREEM tool consists of 49 items which measure aspects of the academic climate. The DREEM scoring tool is divided into five subscales (categorised below) which consist of a set of questions/items and get a separate score. Scores for all five subscales contribute towards the total DREEM score of 196 as follows:

- Students’ perception of learning/teaching (12 questions with a maximum score of 48);
- Students’ perception of teachers/course organisers (11 questions with a maximum score of 44);
- Academic self-perception (8 questions with a maximum score of 32);
- Perception of atmosphere (12 questions with a maximum score of 48); and
- Social self-perception (6 questions with a maximum score of 24).

Modification of the DREEM tool was done by removing question 46, that is ‘my accommodation is pleasant’ (under the subscale social self-perception) since the accommodation of all students from Gauteng and North-West varied enormously during the third year. Hence 49 out of 50 questions were selected (resulting in a maximum DREEM score of 196 instead of 200) and scoring for this subscale (left with 6 instead of 7 questions) was modified in the following way: 0–6 = miserable; 7–12 = not a nice place; 13–18 = not too bad and 19–24 = very good socially. The overall DREEM score was interpreted using the following guidelines: 0–49 = very poor; 50–98 = plenty of problems; 99–147 = more positive than negative; and 148–196 = excellent.^14^

### Data analysis

Data were analysed using Microsoft Excel 2010 and interpreted to draw conclusions about the educational environment in the BCMP programme in the following ways:

- Average DREEM score and average scores for five subscales (North-West + Gauteng).
- DREEM score and subscale scores for both Gauteng and North-West student groups separately.
- Mean scores for all 49 questions – average scores out of 49 and separately for two groups (individual indicators with high and low scores highlighted).
- Total percentage scores: agreement (calculated by adding responses of students who ‘strongly agree’ and ‘agree’ categories); uncertain (calculated by giving a percentage to all student responses which indicated uncertain/unsure); and disagreement (calculated by adding responses of students who ‘strongly disagree’ and ‘disagree’).

Data were scored as per the scoring guide by Roff et al.^14^ All 49 items are in the form of statements pertaining to the student's learning environment. Out of 49, the positive items (such as ‘teaching helps to develop my confidence’) are rated on a 5-point Likert scale scored as 4 for ‘Strongly agree’, 3 for ‘Agree’, 2 for ‘Uncertain’, 1 for ‘Disagree’ and 0 for ‘Strongly disagree’. In addition to that, 9 out of 49 are negative items/worded negatively (such as ‘I find the experience disappointing’) and are reverse scored as 0 for ‘Strongly agree’, 1 for ‘Agree’, 2 for ‘Uncertain’, 3 for ‘Disagree’ and 4 for ‘Strongly disagree’.^14^

Apart from five subscale scores, all 49 items are given an individual mean score out of a maximum of 4 to ‘pinpoint specific strengths and weaknesses’;^15^ ‘Items that have a mean score of 3.5 and over are real positive points’; ‘Items with a mean between 2 and 3 are aspects of the climate which could be enhanced’, and ‘Any items with a mean score of 2 or less need to be examined more closely as they indicate “problem areas”’.^15^ For example, a mean score of 3.5 for a positive statement (‘teaching helps to develop my confidence’) would imply that students positively perceive that teaching develops their confidence, whilst the same score on a negative item (e.g. ‘teachers ridicule the students’) implies that students disagree that teachers ridicule them. Similarly, a mean score of 2 or less for a positive item (e.g. ‘teaching is well focused’) implies that students do not perceive teaching as well focused, whilst the same score on a negative item (I find the experience disappointing) implies that students are not happy with the experience and find it disappointing.

### Ethical considerations

This study received ethics clearance from the Human Research Ethics Committee, Faculty of Health Sciences, University of Witwatersrand, South Africa (Protocol M10802).

## Results

Table 1 shows a summary of scores for both groups along with subscale scores, average DREEM scores and their inference. The average DREEM score for both groups was 130 out of 196, with individual scores of 129 and 131 for Gauteng and North-West students respectively. The subscale which was scored highest by both groups is ‘academic self-perception’ (76.6%), which inferred a feeling more on the positive side, followed by ‘perception of atmosphere’ (66.6%) inferring a more positive attitude. Table 1 further shows that the subscale with a lowest score is ‘social self-perception’ (54.4%), inferring that this aspect of the programme is not too bad.

**TABLE 1:** Average DREEM scores, subscale scores and their inference.

DREEM subscales and maximum scores	Students’ perception of learning		Subscale inference	Students’ perception of teachers		Subscale inference	Academic self-perception		Subscale inference	Perception of atmosphere		Subscale inference	Social self-perception		Subscale inference	DREEM scores
		48		%			44	%			32		%			48	%		24	%		Max. 196
Gauteng third year (*N* = 11)	31	64	A more positive perception	28	63	Moving in the right direction	24	77	Feeling more on the positive side	32	67	A more positive attitude	14	58	Not too bad	129
North-West third year (*N* = 11)	33	69	A more positive perception	31	69	Moving in the right direction	24	76	Feeling more on the positive side	32	66	A more positive attitude	12	51	Not a nice place	131
Average subscale scores for both cohorts and inference	32	66	A more positive perception	29	66	Moving in the right direction	24	77	Feeling more on the positive side	32	66	A more positive attitude	13	54	Not too bad	130

Overall interpretation = More positive than negative.

DREEM, Dundee Ready Education Environment Measure.

Table 2 reflects that the item with the highest mean score in the ‘students’ perception of learning’ subscale is number 21 (‘Teaching helps to develop my confidence’) for the Gauteng group (mean = 3.2) and 16 (‘Teaching helps to develop my competence’) for the North-West group (mean = 3.4). The lowest score (reverse score, mean = 1.3) was given to item 25 (‘Teaching over-emphasises factual learning’) by North-West students. The highest scores in the ‘students’ perception of teachers’ subscale were given to items 32 (‘Teachers provide constructive criticism’) and 37 (‘Teachers give clear examples’) by Gauteng students (mean score 2.9 for both) and item 39 (‘Teachers get angry in class’) by North-West students (reverse score, mean = 3.5). Item 29 (‘Teachers are good at providing feedback to students’) was scored lowest by both groups (mean = 1.4 and 1).

**TABLE 2:** Individual items in the DREEM inventory.

Subscale	Number	Question	Gauteng third year		North-West third year		Average of mean scores a+b/2	Total % of students in Gauteng and North-West third year		
			N1	Mean scores (a)	N2	Mean scores (b)		Agreement %	Uncertain %	Disagreement %
Students’ perception of learning	1	I am encouraged to participate in class	10	3.1	11	3.3	3.2	85.9	14.1	0
	7	Teaching is often stimulating	11	2.1	11	2.6	2.4	54.5	27.3	18.2
	13	Teaching is student-centred	10	2.4	11	2.6	2.5	61.8	19.1	19.1
	16	The teaching helps to develop my competence	10	3.1	11	3.4	3.2	80.9	14.1	5
	20	The teaching is well focused	11	2.6	11	2.6	2.6	63.6	27.3	9.1
	21	The teaching helps to develop my confidence	11	3.2	11	3.0	3.1	86.4	4.5	9.1
	24	The teaching time is put to good use	11	2.5	11	2.8	2.6	72.7	9.1	18.2
	25	The teaching over emphasises factual learning	11	2.0	11	1.3	1.6	45.5	36.4	18.2
	38	I am clear about the learning objectives of the course	11	2.7	11	2.9	2.8	68.2	22.7	9.1
	44	The teaching encourages me to be an active learner	11	2.6	11	2.8	2.7	72.7	22.7	4.5
	47	Long-term learning is emphasised over short-term learning	11	2.5	11	2.9	2.7	68.2	27.3	4.5
	48	The teaching is too teacher-centred	11	2.6	11	2.6	2.6	4.5	36.4	59.1
Students’ perception of teachers	2	The teachers are knowledgeable	11	2.6	11	3.0	2.8	72.7	27.3	0
	6	The teachers are patient with patients	11	2.8	11	3.4	3.1	86.4	4.5	9.1
	8	The teachers make fun of their students	11	2.9	11	3.1	3.0	9.1	9.1	81.8
	9	The teachers are strict and controlling	11	2.8	11	2.7	2.8	9.1	22.7	68.2
	18	The teachers appear to have effective communication skills with patients	11	2.2	11	2.5	2.3	54.5	27.3	18.2
	29	The teachers are good at providing feedback to students	11	1.4	11	1.0	1.2	18.2	13.6	68.2
	32	The teachers provide constructive criticism	11	2.9	11	2.7	2.8	68.2	27.3	4.5
	37	The teachers give clear examples	11	2.9	11	2.9	2.9	81.8	9.1	9.1
	39	The teachers get angry in teaching sessions	11	2.6	11	3.5	3.0	4.5	13.6	81.8
	40	The teachers are well prepared for their classes	11	2.3	11	3.1	2.7	72.7	13.6	13.6
	49	The students irritate and annoy the teachers	11	2.2	11	2.4	2.3	18.2	40.9	40.9
Academic self-perception	5	Learning strategies which worked for me before continue to work for me now	11	2.5	11	2.2	2.4	59.1	9.1	31.8
	10	I am confident about passing this year	11	3.9	11	3.3	3.6	90.9	9.1	0
	22	I feel I am being well prepared for my profession	11	2.5	11	3.0	2.8	63.6	27.3	9.1
	26	Last year's work has been a good preparation for this year's work	11	2.8	11	3.0	2.9	77.3	4.5	18.2
	27	I am able to memorise all I need	11	2.6	11	2.5	2.6	59.1	31.8	9.1
	31	I have learned a lot about empathy in my profession	11	3.4	11	3.5	3.5	95.5	0	4.5
	41	My problem-solving skills are being well developed	11	3.2	11	3.4	3.3	90.9	9.1	0
	45	Much of what I have to learn seems relevant to a career in healthcare	11	3.5	11	3.5	3.5	95.5	4.5	0
Perception of atmosphere	11	The atmosphere is relaxed during ward teaching	11	2.5	11	2.7	2.6	72.7	9.1	18.2
	12	This school is well time-tabled	11	1.7	11	1.7	1.7	40.9	4.5	54.5
	17	Cheating is a problem in this school	11	2.8	11	2.8	2.8	18.2	9.1	72.7
	23	The atmosphere is relaxed during teaching	11	2.9	11	3.2	3.0	90.9	4.5	4.5
	30	There are opportunities for me to develop interpersonal skills	11	3.3	11	2.8	3.0	77.3	18.2	4.5
	33	I feel comfortable in teaching sessions socially	11	3.1	11	2.6	2.9	68.2	22.7	9.1
	34	The atmosphere is relaxed during tutorials	11	2.5	11	3.1	2.8	72.7	22.7	4.5
	35	I find the experience disappointing	11	2.5	11	2.6	2.5	18.2	18.2	63.6
	36	I am able to concentrate well	11	2.6	11	2.5	2.6	63.6	22.7	13.6
	42	The enjoyment outweighs the stress of the course	11	2.5	11	1.6	2.0	36.4	27.3	36.4
	43	The atmosphere motivates me as a learner	11	2.5	11	2.4	2.5	63.6	18.2	18.2
	50	I feel able to ask the questions I want	11	3.5	11	3.4	3.4	95.5	4.5	0
Social self-perception	3	There is a good support system for students who get stressed	11	1.4	11	1.6	1.5	9.1	50	40.9
	4	I am too tired to enjoy this course	11	2.5	11	2.2	2.4	31.8	4.5	63.6
	14	I am rarely bored on this course	11	2.1	11	2.0	2.0	45.5	13.6	40.9
	15	I have good friends in this school	11	3.3	11	2.8	3.0	77.3	13.6	9.1
	19	My social life is good	11	3.0	11	1.8	2.4	59.1	13.6	27.3
	28	I seldom feel lonely	10	1.8	11	1.7	1.8	28.6	14.1	57.3

Items that were scored highest in the ‘academic self-perception’ subscale include number 10 (‘I am confident of passing this year’) for the Gauteng group (mean score = 3.9) and 31 (‘I have learnt a lot about empathy in my profession’) and 45 (‘much of what I have to learn seems relevant to a career in health care’) for the North-West group (mean score = 3.5). Individual items with the highest mean scores in the ‘perception of atmosphere’ subscale include number 49 (‘I feel able to ask the questions I want’) for both Gauteng and North-West, with means of 3.5 and 3.4 respectively, whilst those with the lowest scores include item 12 (‘this school is well time-tabled’) for Gauteng (mean = 1.7) and 42 (‘the enjoyment outweighs the stress of this course’) for North-West students (mean = 1.6). Social self-perception has the maximum items with mean scores of less than 2, namely items 3 (‘there is a good support system for students who get stressed’), 14 (‘I am rarely bored on this course’), 19 (‘my social life is good’) and 28 (‘I seldom feel lonely’).

After the reverse scoring of the 9 negative items (numbers 4, 8, 9, 17, 25, 35, 39, 48 and 49), mean scores of all 49 items for both groups separately and combined were calculated along with per cent agreement, percent uncertain and percent disagreement scores (presented in Table 2). In terms of per cent scores from both groups (indicated as bold in Table 2), 86% of students agree that they are encouraged to participate in teaching sessions, teaching helps to develop their competence (81%) and confidence (86%), the teachers practise a patient-centric approach (86%) and give clear examples during teaching (82%). Almost 90% or more students are confident of passing this year and agree that they have learnt a lot about empathy in their profession (96%), their problem-solving skills are being well developed (91%), they are able to ask the questions they want (96%), the atmosphere is relaxed during lectures (91%), and that much of what they have learnt seems relevant to a career in health care (96%).

### Insight into scores

With regard to mean scores for individual items in the DREEM inventory, 5/49 items for the Gauteng group and 8/49 items for the North-West group were marked at 2 or less than 2 out of the maximum score of 4 (highlighted in Table 2 as bold). Most of these low scores are in ‘social self-perception’ for both groups. In addition to this, 33/49 individual items for both groups in the DREEM inventory have been marked with an average mean score of between 2 and 3, suggesting that some aspects of the BCMP programme could be enhanced. Majority of these middle scores (falling between 2 and 3) are in ‘students’ perception of teachers’ and ‘perception of atmosphere’ for both groups. Lastly, Table 2 reveals that 3/49 individual items for both groups in the DREEM inventory have been marked with an average mean score of 3.5 and above out of the maximum score of 4. These are regarded as ‘real positive points’ for the BCMP programme. All of these high scores are in ‘academic self-perception’.

## Discussion

The educational climate of an institution or course reflects the academic offering and contributes to development of students as practitioners.^16^ Evaluation of the BCMP was done as an exercise to provide initial feedback on the programme. The results provide a profile of students’ perceptions of the BCMP programme, which highlights its strengths and weaknesses. Whilst the BCMP programme is new and thus still undergoing modification, this evaluation indicates that it is positively perceived by students.

The DREEM evaluation of the Wits BCMP programme shows an average score of 130/196 (with minor differences between North-West and Gauteng students), indicating a ‘more positive than negative’ educational environment. The global DREEM score reported by medical schools in countries such as the United Kingdom (124/200),^15^ India (123/200),^17^ Sri Lanka (108/200),^18^ Nigeria (118/200)^1^ and Iran (100/200)^19^ were lower than in our study. The differences could be attributed to the fact that our total DREEM score was based on a single cohort of Clinical Associate students, whilst other studies either administered the DREEM questionnaire to a large number of undergraduate students at different years of enrolment,^17^ administered the DREEM questionnaire to all students in medical schools,^15^ or conducted comparative cross-sectional studies in a number of medical schools.^20^

There are other studies with a single cohort of students like our study which also had a lower DREEM score of 106/200^21^ (Kuwait) and 118/200 (Malaysia).^22^ The differences could also be attributed to the fact that the BCMP is a new programme which makes a learning environment exciting for Clinical Associates. Our DREEM score was similar to that found in a study conducted amongst medical schools in Nepal (130/200).^23^

The results demonstrate a positive perception of learning by the students, which reflects that the BCMP offers a favourable learning environment (32/48). The highest individual scores in this section indicate that teaching helps to develop the students’ confidence (3.2) and competence (3.1), which are important traits needed in their future professional settings. Students also believed that they were encouraged to participate in class (3.2), which is an indication of an open and interactive learning atmosphere. Contrary to our findings, a study conducted in Malaysia with second-year medical sciences students (*n* = 67) found a mean score of 1.88, which indicates that teaching does not provide enough experiences to help them to have confidence.^22^

One of the areas of concern perceived by students in the current study is that ‘teaching over-emphasises factual learning’ (45.5%). This finding is consistent with that of Arzumanet al.^22^ We were unsure whether, whilst answering this question, students referred to class-based teaching (which includes factual learning) or hospital-based teaching (which is more of a hands-on experience). Hence due to lack of clarity the findings cannot be interpreted to draw a conclusion as to whether the BCMP programme stressed factual learning. Further exploration in this regard through qualitative research can provide a clear picture.

Two more results from this section need to be addressed. Only 54.5% of students agreed that ‘teaching is often stimulating’, and only 61.8% agreed that ‘teaching is student-centred’, with mean scores of 2.4 and 2.5 respectively. The latter is important as the BCMP curriculum focuses on student-centred learning and problem-solving. More emphasis in the curriculum on promoting student-centred learning is suggested.

The next area of the study demonstrates that educators in the BCMP programme are being perceived as moving in the right direction in terms of quality of their teaching. Students in the North-West group were more satisfied with their teachers than the Gauteng group. Best traits of teachers as perceived by students include providing constructive criticism, giving clear examples in class and not getting angry at students. This is encouraging for the BCMP programme, as the findings of a study conducted by Aghamolaei and Fazel^19^ were that student perceptions of teachers were that they do not provide constructive criticism.

Our study highlights two areas that need to be addressed for this section. Results indicate a need for teachers to improve their communication with patients. This finding is in contrast with Arzuman et al.'s^22^ study, which found that students indicated that their teachers had good communication skills. The greater area of concern is that only 18.2% agreed with the statement ‘teachers are good at providing feedback to the students’ (1.2), with 68.2% disagreeing with the statement. This question had the lowest mean score of the study. The understanding of the term ‘feedback’ may not be clear in this context. We are not sure if students referred to timely feedback in class, feedback on return of test marks, periodic feedback on their assignments and performance in exams and in clinics, including lack of clarity on the person who provided feedback (teachers in class, clinical mentors/tutors/course organisers). This area must be explored further through qualitative research for a better understanding, with steps being implemented to improve the process of teachers providing feedback.

Different to studies conducted in traditional and innovative medical schools, which found that academic self-perceptions and social self-perceptions were rated lower,^24^ in our study ‘Academic self-perception’ was marked highest by both groups (24/32). Students strongly believed and were confident that they would pass (3.6, highest mean score of the study), they have learnt a lot about empathy in their profession, and much of what they have learnt seems relevant to a career in healthcare. They also perceive that their problem-solving skills are being well developed, which is an important objective of the BCMP programme and indicates that the learning environment is contributing to fulfilling the course objectives. The students’ perceptions were proved accurate, because they all passed the course and are now working as Clinical Associates. The findings of our study differ from those of Hamid et al.,^25^ who found that the subscale with the highest mean score was ‘students’ perception of learning’ (27/48), and ‘academic self-perception’ had a lower score of 20/32. Our study reveals a more positive feeling amongst the students.

All students perceived their ‘learning atmosphere’ in the category of ‘a more positive attitude’, with a slightly higher score for Gauteng students compared to North-West students. The best perceived aspect of the learning atmosphere is that students from both groups were able to ask questions they wanted, which potentially enhanced their communication skills with and learning from teachers. The atmosphere was relaxed during teaching, thereby promoting teacher-student interaction and sharing of scientific and conceptual knowledge. Other studies also found that the overall learning atmosphere for the students was comfortable.^22^

Areas of concern included perceptions by both student groups that the course was not well time-tabled (1.7), which may be explained by the fact that the BCMP was newly introduced and undergoing modifications. Clearly the results indicate an area for coordinators to address. Similarly, other studies found that students’ perceptions of atmosphere were that the school/course was not well time-tabled.^19,21^ North-West students perceived that the course was too stressful for them to enjoy, which could be attributed to their placements in distant areas far from family and friends.

Although all of the students had different social environments at their places of clinical placements, their overall ‘social self-perception’ does not stand as an indicator with good scores. Students from Gauteng perceived their social environment as ‘not too bad’, whilst those from North-West perceived it as ‘not a nice place’ – which could be attributed to distant location and being away from home for a long time. A positive perception was that students had good friends within the groups, indicating that fellow students acted as supporters. This is similar to the findings by Arzuman et al.,^22^ who found that students had a good social life, which was reflected by them having good friends on campus. Similar to other studies,^19^ areas of concern include lack of good support systems for students who get stressed (mean score of 1.5, second lowest score), feeling lonely and lack of a good social life. It was unclear as to what type of support system they referred to (academic/social/personal). In order to improve the social aspect and ensure that apart from academics students also enjoy their social life, we need to further explore this aspect through qualitative research and take measures accordingly.

Considering the nature of the BCMP, which is an innovative curriculum and combines both classroom- based learning as well as external hospital/clinic-based teaching, DREEM does have some limitations in this context. Terms such as ‘course organisers/teachers’, ‘atmosphere’ and ‘learning/teaching’ present ambiguity in terms of whether the students perceived these for classroom- or hospital-based learning environments. In this regard we are considering designing instruments that are suited to evaluate the learning environment at different stages of the BCMP degree, so that they can capture students’ perceptions without ambiguity. The venue used to administer the questionnaire might have influenced the students’ responses about the programme.

## Conclusion

The DREEM evaluation study offers a preliminary introspection into the learning environment of the BCMP programme. Despite the sample size, this study tried to evaluate the overall educational climate of the innovative BCMP curriculum at Wits. Results from this study demonstrated that the BCMP programme was perceived as a positive learning environment, contributing to the course objectives. This study highlighted strengths and weaknesses in the programme that can guide course organisers to design/modify the course.

As this was the first cohort, future evaluations will need to be conducted periodically on a larger scale with an increased sample size and incorporating more variables, and a modified questionnaire better suited to the context of the BCMP learning environment, with an additional qualitative component for better exploration of students’ perceptions.
